# The Hepatitis B Vaccine and Celiac Disease: More Lights Than Shadows?

**DOI:** 10.5812/hepatmon.7878

**Published:** 2013-01-20

**Authors:** Giovanna Vitaliti, Angela Lanzafame, Mario La Rosa, Salvatore Leonardi

**Affiliations:** 1 Department of Pediatrics, University of Catania, Catania, Italy

**Keywords:** Celiac Disease, Hepatitis B Virus, Vaccines, Injections, Intramuscular

Dear Editor,

Actually, in literature, it is known that HLA-DQ2 haplotype is over-represented in celiac population and since both HBsAg protein fragments and gliadin peptides bind to HLA-DQ2 molecules; their competition may favors a defective antibody response against the recombinant HBsAg vaccine in active celiac disease (1). On the other hand, some studies demonstrate that in celiac individuals, the gluten intake at the time of vaccination influences the vaccine-induced immune response (2). All these hypotheses are still argument of debate because the non-responsiveness of celiac patients to the HBV vaccine remains a significant public health problem, although there is no convinced evidence that patients with viral hepatitis B show an increased risk of celiac disease (3). It would be important to assess the possible vaccination strategy in order to reduce this potential “healthy-reservoir” of infection. Regarding to this, new vaccination strategies for celiac patients were proposed in literature: the first one was the use of booster doses of HBV vaccine by intra-muscular route (IM), using higher doses or a higher number of injections after a gluten free diet; the second was the performance of vaccine boosters by intra-dermal route (ID) (4). The rational of the second strategy is that as opposed to intramuscular vaccination (IM), which relies on a T-cell mediated response, the vaccine directly introduced into the skin activates a dendritic-cell-mediated immune response, using a lower dose of antigen (5). The comparison of both routes suggests that both ID and IM routes are effective options to administer a booster dose of HBV vaccine in celiac patients although the ID route seems to result in a higher percentage of patients with an anti-HBs titer > 1000 IU/L (6). A recent retrospective cost-benefit analysis of ID hepatitis B vaccination reported a cost reduction exceeding 50% in comparison with a standard IM vaccine regimen (7). In fact the increase of anti-HBs antibodies is satisfactory after ID injection in all patients, a lower dose of the vaccine can be used, the cellular immune responsiveness to HBsAg can be easily assessed by the development of a skin reaction at the injection site (8) and thanks to the setup of this reaction there is no need of venous withdraws to test the serum anti-HBs concentration after the booster dose. On this debate, few shadows remains unsolved, because it is still not clear if celiac non-responders show an immunity energy since birth and do not respond since the first dose of vaccination or they lose their antibody protection with the flow of time, as physiologically happens in normal people, even if in a shorter period of time. Consequently, two subgroups of non-responders should be classified: the first group is the "pure non-responders", that are celiac patients with a non-responsiveness since the first booster dose of HBV vaccine, and the second is the group of patients that lost their memory after a period of time after the vaccine injection. On this regard, in literature it is also demonstrated that immunological memory persists more than 10 years after the immunization of infants and adolescents with a primary course of vaccination (9) and there is general consensus that successfully vaccinated people who have lost their antibodies years after primary vaccination usually show a rapid anamnestic response when boosted (9). This means that the immunological memory for HBsAg can outlast antibody detection, providing long-term protection against the disease and the development of the carrier state. Therefore, until new data shed light on this issue good advice seems to be:

1) In consideration of the possible relationship between anti-HBs titers and compliance with gluten free diet (GFD), they should be always revaccinated after the decrease of specific celiac antibodies, which usually occurs after about 1 year of a strict GFD in order to maintain a high level of immunization.

2) After all, patients with CD should always receive a booster dose of vaccine every 10 years independently by their status of “pure” unresponsiveness.

3) HBV vaccine by intradermal route should be preferred instead of intramuscular route because of the use of lower doses of vaccine and the possibility to assess cellular immune responsiveness to HBsAg by the development of a skin reaction at the injection site (8).
